# Gonorrhœa and Homœopathy—A Defence of Dr. T. F. Allen

**Published:** 1891-01

**Authors:** J. C. White

**Affiliations:** Portchester, N. Y.


### GONORRHŒA AND HOMŒOPATHY.—DEFENCE OF DR. T. F. ALLEN.

J. C. White, M. D., Portchester, N. Y.

His critics are not wanting in numbers, but they appear to me to be wanting in a true and most reasonable interpretation of his remarks. In verbal discussions it is comparatively easy to look at a subject from the same standpoint, while in written statements it is sometimes difficult to do so. Again, a section of given remarks may bear a very different import when abstracted from that which preceded and that which followed it. Would his critics think of making subdivisions in the classification of the so-called allopathic physician other than good, better, best, or poor, poorer, and poorest? We see nothing wanting in his own explanation to those of brotherly feelings in the profession.

Dr. Wells has given us, in his definition of a homœopathic physician, the "highest type"—something to be obtained. Hence the "School," as we are called—denoting learning—progression. If none are homœopathic physicians who fail to give the simillimum in the minimum dose in each case I fear we may have to resort to the method of the "Methodist Christian" denomination—*i. e.*, receive all applicants to our society on probation, and a long probation it would be. Dr. Wells himself represents his own standard as well as any physician of our acquaintance. In a recent number of THE HOMŒOPATHIC PHYSICIAN he recommends the "Bœnninghausen powders" indiscriminately in cases of croup. If we are to be judged by the "Law," this certainly is a deviation from it, and the 200th dilution is no apology for the deviation.

We do not mention this in a spirit of retaliation or unkindness, but to show how very natural it is for each and every physician to be governed by "clinical experience"—the method on which the practice of medicine was first founded.

"Let him who is without sin cast the first stone."

Highly diluted medicines given entirely upon a clinical basis is what I understand Dr. Allen to mean by "High Potency Allopathy." It is like the old school, "experimental medicine," and I do not feel like quarreling with him for the statement.

True, we may get some light in this way. The utility and success of the method depends entirely upon the value of clinical symptoms. Hahnemann when using the method did it guardedly and with the lower dilutions—as it were, "feeling" for confirmations rather than adopting them as a basis for prescriptions. I agree with Dr. Allen and many others who have but little confidence in the clinical part of our repertory. I beg the patience of our readers while I give my reasons for this statement. *It is only* when a *given* medicine sensibly and perceptibly *relieves or modifies* the *intensity* of symptoms which are *expressive* of *disease* or of *functional disturbance* that it can *acquire clinical significance.*

The fact that a case of Dr. A.'s recovered from typhoid fever under a given medicine, and the case of Dr. B.'s recovered from diphtheria or scarlatina under another given medicine, etc., is comparatively of no clinical importance. I have learned to have too much faith in the *vis medicatrix naturæ* to believe it.

The old-school physicians have but little faith in medicine, and expect nothing more than palliative and sustaining results from it. There are some exceptions to this statement, while I observe that homœopathic physicians call every resolution from acute disease while using their medicines a *cure.*

I was the only resident physician of a small country town during a period of twenty-one years. I served as "family physician" over seven hundred resident families. I seldom passed a house on my professional rides that I did not so serve. (I make this statement to prove conclusively that my cases were not selected ones.) The first twelve years of my service I practiced allopathy exclusively, the following nine years there and the subsequent four years here I have practiced Homœopathy "as well as I could." During the first eighteen years of practice I did not lose one case of uncomplicated pneumonia—by uncomplicated I mean those not suffering from heart disease, hereditary disease of lungs, or extreme old age. It would be arbitrary to say that I *cured* each case of the subsequent six years and not those occurring during the previous twelve years. It is but just to say that in those cases of recovery resolution commenced with the administration of the remedy, while in others effusion and absorption went on by nature's method. How much was done in these cases by measures addressed to sustaining nutrition is difficult to say. In every apparently serious case measures which Dr. Allen calls "other than homœopathic" were employed, such as warm fomentations; also counter-irritation, such as mustard plasters or dry cupping in the first stage of disease, and subsequently warm poultices, often enveloping the whole chest.

Often have I left a patient, who on my first visit was suffering from dyspnœa in the first stage of pneumonia (congestion), comparatively comfortable after a thorough *dry cupping.* I used common tumblers, exhausting the air by a common taper. Now, after learning the value of Aconite in this condition, I doubt if the disease can be so far aborted by it alone. I now trust it or the indicated remedy alone when symptoms are not grave or violent. When they are so I dare not omit such adjuvants as have served me well in extremes. While I cannot depreciate the value of medication, I affirm that pneumonia of average severity may recover without it, and that a very large per cent. of recoveries are not *cures.*

While practicing allopathy I had occasion to call upon a homœopathic physician, residing and practicing in a country place, about six miles distant. He was a man much respected, intelligent, and an enthusiastic homœopath—as every physician should be. In course of conversation he asked me if I had seen much typhoid during the season. In reply I stated that I had treated but two cases, at which he expressed surprise, adding that he had treated more than forty cases! Of course it was my turn to express surprise, knowing thoroughly the sanitary condition of the country around me—visiting each section frequently. The improbability of the statement amounted to a certainty with me. Noticing my apparent skepticism, he remarked that but one or two developed the real typhoid, but that all would have done so but for his treatment; that he cured them at the very beginning! I do not doubt but that the fever may be cured by correct medication at the outset, at least so arrested that its characteristic lesions are not developed. But his limited practice in a healthy country district makes his statement *most improbable.*

I mention this, in point, to question the value of clinical symptoms given by the profession as a basis for prescription.

Dr. T. F. Allen declares, "And it is absolutely true that every physician in large practice is obliged to use other than homœopathic methods in the treatment of the sick." A critic of this remark, with, perhaps, a comfortable office practice of chronic cases would hardly feel the truth and practicability of this assertion. Custom cannot make the *adjuvants* (which we all use) to the application and the administration of the homœopathic remedy—essentially "homœopathic methods." The application of warm fomentations—emollient poultices—and even minor surgery is often admissible at times where it is not really essential to a cure. If they are helpful—palliative—the common sense of the patient demands them and we must use them. I have but just returned from a patient who had failed to "hit the nail on the head" and bruised his thumb badly. The nail was black and the blood-pressure so great, though a strong man, he was convulsed with pain. The administration and the application of Arnica would have relieved him of pain in time, but the application of the knife, letting out the effused blood, relieved him in one minute. To use his own language, "he was in heaven." I consider that I should have been culpable had I left him with medicine only. Yet, such measures are "other than homœopathic," "not for their *cure* but for their palliation."

Dr. E. W. Berridge, in quoting Dr. T. F. Allen, says: "Dr. T. F. Allen's argument is that in incurable cases Homœopathy is insufficient." He adds, "On the contrary I have found by experience that Homœopathy relieves these cases and promotes euthanasia far better than allopathy."

All earnest students and practitioners of Homœopathy will indorse Dr. Berridge's statement.

All such students and practitioners who know Dr. T. F. Allen, and his resources, believe him to be the last man to resort to methods other than homœopathic in such cases. I read his argument, that there are exceptions to the rule, and that, not because of any "wrong teaching of Hahnemann," but because Hahnemann *lived* and *wrought* in the *present century*, and that our materia medica is consequently so imperfect that we can not meet all indications of disease successfully. I cannot recall but two incurable cases in my later practice where I felt obliged to resort to "other methods." Those were of chronic Bright's disease—a most distressing disease to die of. I will add that this deviation in one case was indorsed by two of the most able and enlightened homœopathic physicians of my acquaintance. We all have our "book cases;" they read beautifully, and illustrate and confirm the great truth of Homœopathy, but I confess that my own failures to cure chronic cases would fill more pages, and perhaps our failures, if we were brave enough to report them, would be quite as instructive as our cures. I am persuaded that to be successful in chronic cases, one must have patients who have some knowledge or experience of homœopathic methods, of which we have comparatively few in country towns. Although we explain our method, they have not the faith which gives patience to wait results. The severe and sometimes terrible aggravations which frequently follow the administration of the highest dilutions often frighten them away. I do not now use them except when the medium dose fails to complete the cure. Dr. Allen has, apparently, offended some of the profession by his expression of preference for the lower dilutions. I must confess that I find nothing wanting in them in the treatment of a great majority of acute cases. I find that the experience of all of my acquaintances in the profession. In the matter of dose or dilution I think we may very properly be governed by clinical experience.

---

